# Dissociated neuronal phase- and amplitude-coupling patterns in the human brain

**DOI:** 10.1016/j.neuroimage.2020.116538

**Published:** 2020-04-01

**Authors:** Marcus Siems, Markus Siegel

**Affiliations:** a Centre for Integrative Neuroscience, University of Tübingen, Germany; b Hertie Institute for Clinical Brain Research, University of Tübingen, Germany; c MEG Center, University of Tübingen, Germany; d IMPRS for Cognitive and Systems Neuroscience, University of Tübingen, Germany

**Keywords:** MEG, Functional connectivity, Neuronal oscillations, Phase-coupling, Amplitude-coupling, Synchrony, Attenuation correction, Human connectome project

## Abstract

Coupling of neuronal oscillations may reflect and facilitate the communication between neuronal populations. Two primary neuronal coupling modes have been described: phase-coupling and amplitude-coupling. Theoretically, both coupling modes are independent, but so far, their neuronal relationship remains unclear. Here, we combined MEG, source-reconstruction and simulations to systematically compare cortical amplitude-coupling and phase-coupling patterns in the human brain. Importantly, we took into account a critical bias of amplitude-coupling measures due to phase-coupling. We found differences between both coupling modes across a broad frequency range and most of the cortex. Furthermore, by combining empirical measurements and simulations we ruled out that these results were caused by methodological biases, but instead reflected genuine neuronal amplitude coupling. Our results show that cortical phase- and amplitude-coupling patterns are non-redundant, which may reflect at least partly distinct neuronal mechanisms. Furthermore, our findings highlight and clarify the compound nature of amplitude coupling measures.

## Highlights

• Systematic comparison of cortical phase- and amplitude-coupling patterns.
• Demonstration of genuine amplitude coupling independent of phase coupling bias.
• Amplitude- and phase coupling patterns differ across many cortical regions and frequencies.

## 1 Introduction

The brain is a distributed information processing system. Correlated oscillations of neuronal activity have been proposed to facilitate and orchestrate communication between distant brain regions (Fries, 2015; Siegel et al., 2012; Singer, 1999). In this context, neuronal firing is described as a probabilistic process that is shaped by the phase and amplitude of oscillatory rhythms (Destexhe et al., 1999; Engel et al., 2013, 2001; Fries, 2015; Hillebrand et al., 2012; Hipp et al., 2012; Jahnke et al., 2014; Jensen and Mazaheri, 2010; Siegel et al., 2012). When temporally correlated, co-fluctuations of local oscillations may enhance effective communication between neuronal populations and enable the multiplexing of neuronal information (Akam and Kullmann, 2014; Lopes da Silva, 2013; Singer, 2013). There are two primary coupling modes between neuronal oscillations: phase-coupling and amplitude-coupling (Bruns et al., 2000; Siegel et al., 2012; Engel et al., 2013).

Phase-coupling refers to a consistent phase-alignment between neuronal oscillations, which may reflect a frequency specific signature of neuronal interactions (Siegel et al., 2012). Moreover, phase-coupling may itself modulate effective connectivity by aligning rhythmic excitability fluctuations to rhythmic spike inputs (Fries, 2015). Consistent with this functional role, long-range neuronal phase-coupling reflects various cognitive processes, such as e.g. selective attention (Bosman et al., 2012; Buschman and Miller, 2007; Gregoriou et al., 2009; Siegel et al., 2008), perception (Hipp et al., 2011), memory (Fell and Axmacher, 2011; Palva et al., 2010) and task switching (Buschman et al., 2012). Moreover, task-dependent phase-coupling is expressed in well-structured, large-scale cortical networks (Hipp et al., 2011; Marzetti et al., 2019; Palva et al., 2010).

Amplitude-coupling refers to the temporal co-modulation of the amplitude (or power) of neuronal oscillations. Like phase-coupling, amplitude-coupling may not only result from, and thus reflect, neuronal interactions, but may also regulate these interactions by temporally aligning distant processes associated with fluctuating oscillations (Siegel et al., 2012; von Nicolai et al., 2014). Also amplitude-coupling is expressed in well-structured cortical networks that match known anatomical and functional connectivity (Hipp et al., 2012; Siems et al., 2016), resemble fMRI correlation patterns (Brookes et al., 2011; Deco and Corbetta, 2011; Destexhe et al., 1999; Hipp and Siegel, 2015; Mantini et al., 2007; Nir et al., 2008; O’Neill et al., 2015), and are more stable than phase-coupling networks (Colclough et al., 2016; Wang et al., 2014). Amplitude-coupling is largely driven by amplitude dynamics below 0.1 ​Hz (Hipp et al., 2012), which may reflect the slow establishment and decay of communicating networks (Destexhe et al., 1999; Leopold et al., 2003; Mantini et al., 2007; Larson-Prior et al., 2011; Hipp et al., 2012; Engel et al., 2013).

Both coupling modes may provide versatile biomarkers for various neuropsychiatric diseases (Fornito et al., 2015; Stam, 2014) including autism (Kitzbichler et al., 2015), schizophrenia (Cetin et al., 2016; Maran et al., 2016), epilepsy (Burns et al., 2014; van Dellen et al., 2014; Zerouali et al., 2016), dementia (Koelewijn et al., 2017; Maestú et al., 2015), Parkinson’s disease (Oswal et al., 2016), multiple sclerosis (Cover et al., 2006; Schoonheim et al., 2013; Tewarie et al., 2014) and blindness (Hawellek et al., 2013).

Despite the strong interest and rapidly growing evidence on both, neuronal phase- and amplitude coupling measures, their relationship remains unclear. On the one hand, both coupling-modes could be independent. There could be phase-coupling without amplitude-coupling and vice versa (Siegel et al., 2012). In this case, phase- and amplitude-coupling could be caused by distinct neuronal mechanisms and their cortical coupling-patterns may be dissociated (Daffertshofer et al., 2018). On the other hand, both coupling modes may be tightly linked, e.g. if both modes reflect the same underlying neuronal interactions, or if one coupling mode causes the other (von Nicolai et al., 2014; Womelsdorf et al., 2007). In this case, the cortical patterns of both coupling modes may be highly similar or even identical. Intermediate scenarios are also possible. The central aim of this study was to non-invasively investigate this relationship between phase- and amplitude coupling in the human brain with MEG.

Addressing this questions is complicated by a methodological peculiarity of the estimation of amplitude coupling that has recently been pointed out (Palva et al., 2018). If erroneous coupling due to field-spread is suppressed by orthogonalization (Brookes et al., 2012; Hipp et al., 2012), measures of amplitude coupling are also partially sensitive to phase coupling (Palva et al., 2018). In other words, the measured amplitude-coupling reflects a mixture of the genuine amplitude-coupling of interest and spurious amplitude-coupling due to phase-coupling.

Thus, we approached our central question in two steps. First, we tested if there is a genuine component to the cortical amplitude-coupling measured with MEG, beyond the spurious amplitude-coupling induced by phase-coupling. Second, we addressed our main question how phase- and genuine amplitude-coupling relate. To this end, we systematically compared the cortical correlation structure of both coupling modes across the human brain.

## 2 Materials and methods

### 2.1 Subjects and dataset

We analyzed resting-state MEG measurements from 95 subjects included in the publicly available human connectome project (HCP) S900 release. Participants were healthy adults in the age range between 22 and 35 (n_22-25_ ​= ​18, n_26-30_ ​= ​40, n_31-35_ ​= ​37). The sample included 45 females. The resting-state measurements included up to three 6-min blocks with short breaks in between measurements. Data were recorded with a whole-head Magnes 3600 scanner (4D Neuroimaging, San Diego, CA, USA) situated in a magnetically shielded room (for further details see: Larson-Prior et al., 2013). Additionally, subjects were scanned on a Siemens 3T Skyra to acquire structural T1-weighted magnetic resonance images (MRI) with 0.7 ​mm isotropic resolution (Van Essen et al., 2013).

### 2.2 Data preprocessing

We used the preprocessed data as provided by the HCP pipeline (Larson-Prior et al., 2013). This includes removal of noisy and bad channels, bad data segments and physiological artifacts by the iterative application of temporal and spatial independent component analysis (ICA) (Larson-Prior et al., 2013; Mantini et al., 2011).

### 2.3 Physical forward model and source modeling

MEG sensors were aligned to the individual anatomy using FieldTrip (Oostenveld et al., 2010). We segmented the individual T1-weighted images and generated a single shell head model to compute the physical forward model (Nolte, 2003). We computed the forward model for 457 equally spaced (~1.2 ​cm distance) source points spanning the cortex at 0.7 ​cm depth below the pial surface (Hipp and Siegel, 2015). This source shell was generated in MNI-space and non-linearly transformed to individual headspace. Source coordinates, head model and MEG channels were co-registered on the basis of three head localization coils.

The sensor-level MEG data was projected to source space using linear beamforming (Gross et al., 2001; Van Veen et al., 1997). This spatial filtering approach reconstructs activity of the sources of interest with unit gain while maximally suppressing contributions from other sources.

Coordinates for the seed-based connectivity analyses were adopted from Hipp et al. (2012). For every seed, the source location of the 457 shell positions with minimum Euclidean distance from the seed coordinates was chosen: left auditory cortex (lAC) [-54, −22, 10]; left somatosensory cortex (lSSC) [42, −26, 54]; medial prefrontal cortex (MPFC) [-3, 39, −2] (all MNI coordinates).

### 2.4 Spectral analysis

Time-frequency estimates of the time-domain MEG signal were generated using Morlet’s wavelets (Goupillaud et al., 1984). The bandwidth of the wavelets was set to 0.5 octaves (1 spectral standard deviation) with a temporal step-size of half the temporal standard deviation. We derived spectral estimates for frequencies from 1 to 128 ​Hz in quarter octave steps.

### 2.5 Coupling measures

We estimated amplitude coupling using amplitude envelope correlations of orthogonalized signals (Hipp et al., 2012). Volume conduction effect were discounted by orthogonalizing the two complex signals at each point in time before correlation (Brookes et al., 2012; Hipp et al., 2012):

$$y_{orth}\left(t,f\right)=imag\left(y\left(t,f\right)\frac{x\left(t,f\right)’}{\left|x\left(t,f\right)\right|}\right)$$

The *imag* operator describes the imaginary part of the signal. The complex signals *x* and *y* are a function of time and frequency. *x*’ is the complex conjugate of *x*. Discounting volume conduction with orthogonalization is only optimal for data with a Gaussian distribution (Brookes et al., 2014). Finally, we computed the Pearson correlation between the logarithm of power envelopes of the signals *x* and *y*_*orth*_.

As a measure of phase coupling we applied the weighted phase lag index (wPLI; Vinck et al., 2011). The wPLI takes only the imaginary part of the cross-spectrum into account and normalizes it with the average absolute imaginary contribution within the time series.

$$wPLI=\frac{\left|mean\left(imag\left(C_{x,y}\right)\right)\right|}{mean\left(\left|imag\left(C_{x,y}\right)\right|\right)}$$

$$C_{x,y}=xy’$$

Here, *C*_*x,y*_ is the cross-spectrum between the two complex signals *x* and *y* defined as the product of *x* and the complex conjugate of *y*. The imaginary part of the cross-spectrum is insensitive to volume conduction since it has no contribution from zero phase lagged parts of the signal (Nolte et al., 2004; Vinck et al., 2011). We computed both coupling measures for the full correlation matrices for all subjects and frequency bands.

### 2.6 Data simulation

Palva et al. (2018) showed that amplitude correlations based on orthogonalized signals yield spurious correlations, given a consistent non-zero phase delay between signals. We employed the simulation approach put forward by Palva et al. (2018) as a generative model to estimate these spurious correlations. We computed a model for every connection, subject and frequency using empirical values for the free parameters. With this approach, we generated complete correlation matrices for every subject and frequency to estimate the spatial patterns of spurious amplitude-coupling. We modeled every two signals *x* and *y*

$$x=A_{x}\left(t\right)e^{ip_{x}\left(t\right)}+mA_{y}\left(t\right)e^{i(p_{y}\left(t\right)+s_{x,y})}$$

$$y=A_{y}\left(t\right)e^{i(p_{y}\left(t\right)+s_{x,y})}+mA_{x}\left(t\right)e^{ip_{x}\left(t\right)}$$

where *A(t)* and *p(t)* are vectors representing the amplitude and the phase of the sources, respectively. In analogy to volume conduction, the source data is linearly mixed by the parameter *m*. This value is determined from the empirical data as the multiplication of the filter matrix *F*_*x,f*_ with the leadfield *L*_*y*_ (i.e, the resolution matrix) projected onto the first principal dipole direction *P1* at *x*.

$${m\left(f\right)}_{x,y}=F_{x,f}L_{y}P1_{x}$$

For every connection, we computed the model in both directions. *s*_*x,y*_ is the phase shift between the two signals and was set to the estimated empirical phase shift for every connection, frequency and subject (see 2.7 below).

We determined the amplitude *A(t)* vectors as follows:

$$A_{x}\left(t\right)=\left|F\left(n_{1}\left(t\right)+c_{A}n_{2}\left(t\right)\right)\right|$$

$$A_{y}\left(t\right)=\left|F\left(n_{2}\left(t\right)+c_{A}n_{1}\left(t\right)\right)\right|$$

where *n*_*1*_*(t)* and *n*_*2*_*(t)* are vectors of normally distributed random numbers with data length of 300 ​s, a pink spectrum and a sampling frequency of 400 ​Hz approximately matching the original data (Larson-Prior et al., 2013). The || operator refers to the modulus. *c*_*A*_ denotes the amplitude coupling between the sources *x* and *y*, which was set to 0. The function *F* is the complex wavelet transformation of the vectors at the frequency of interest. The wavelet transformation parameters matched our analysis of the empirical data (see above). Analogously, we generated the phase *p(t)* vectors:

$$p_{x}\left(t\right)=angle\left(F\left(n_{3}\left(t\right)+c_{p}n_{4}\left(t\right)\right)\right)$$

$$p_{y}\left(t\right)=angle\left(F\left(n_{4}\left(t\right)+c_{p}n_{3}\left(t\right)\right)\right)$$

where *n*_*3*_*(t)* and *n*_*4*_*(t)* are again 300 ​s vectors of normally distributed random numbers with a pink spectrum at a sampling frequency of 400 ​Hz. All 4 *n*-vectors were drawn anew for every connection and simulation run (see below). *c*_*p*_ denotes the phase-coupling and was set to the estimated empirical phase-coupling for every connection, frequency and subject (see 2.7 below).

Finally, we computed the amplitude coupling of the orthogonalized signals *x* and *y* (see above) to quantify the strength of amplitude coupling (*AC*_*spur*_) that would be expected given the empirical parameters and no ground truth amplitude coupling (*c*_*a*_ ​= ​0). We computed the full correlation matrices for every subject and frequency. To reduce the variance induced by finite sampling, we averaged the correlation matrices across ten simulations. Furthermore, we averaged the two directions of orthogonalization, *x* on *y* and *y* on *x*, in the final correlation matrices. We correlated the spatial patterns *P*_*ACspur*_ of spurious amplitude coupling with the spatial patterns of empirically measured amplitude coupling *P*_*ACmeas*_ to quantify the similarity of these spatial patterns.

Finally, we orthogonalized the measured amplitude coupling patterns to the spurious amplitude coupling patterns by linear regression to estimate the corrected amplitude coupling patterns.

### 2.7 Estimated empirical phase shift and phase coupling

The wPLI quantifies phase-coupling but is not identical to the phase-coupling *c*_*p*_ employed in the above simulations. In other words, if one simulates two signals with *c*_*p*_ set to an empirically measured wPLI the simulated signals will have a wPLI that is different from the empirically measured wPLI. The same applies to the phase shift *s*_*x,y*_ between the two signals. Thus, for each connection, frequency and subject, we employed the following approach to compute the estimated empirical phase shift and phase coupling that, when used in the above simulation, yielded a wPLI and measured phase shift between the simulated signals that matched the empirically measured wPLI and phase shift.

We performed the simulation described above (see 2.6) 800,000 times covering the entire space of possible mixings, phase couplings and phase shifts: mixing *m* between 0 and 1 (0.01 steps), phase shift *s*_*x,y*_ between 0 and π (π/100 steps) and phase coupling *c*_*p*_ between 0 and 0.8 (0.01 steps). For each parameter combination, we computed the phase shift, i.e. the angle of the mean complex coherency, and the wPLI of the simulated signals. To stabilize these estimates, we averaged these values across 1000 repetitions of the simulations. For any connection with a given mixing *m*, we then determined which combination of *c*_*p*_ and *s*_*x,y*_ yielded the empirically measured wPLI and phase shift of the simulated signal. We then employed this estimated empirical phase shift and phase coupling for each connection at hand (see Fig. S1 for an example and assessment of the estimation quality).

### 2.8 Reliability estimation

To compare the reliability, i.e. reproducibility, of functional connectivity measures, we correlated the seed connectivity-patterns. We first averaged the correlation matrices acquired in the three runs of each subject before correlating pairwise between subjects (between-subjects reliability *rel*_*bs*_).

$$rel_{bs,M1,M2,s1,s2,f}=corr\left(P_{M1,s1,f},P_{M2,s2,f}\right)$$

where *M1 and M2* denote the different connectivity measures (*AC*_*spur*_, *AC*_*meas*_, or *PC*). A pattern *P* describes the connectivity of a given seed with the rest of the source-model, i.e. one column of the full correlation matrix. s1 and s2 denote the subjects involved in the computation, where *s1 ≠ s2*. All reliabilities were independently computed as a function of frequency *f*.

Only reliable signals can be correlated and corrected for attenuation (see below). Therefore, we statistically tested for reliabilities larger than zero (one-sided *t*-test, df ​= ​94 (n ​= ​95), FDR correction, see below) and excluded connections with non-significant reliability. For measured amplitude coupling, spurious amplitude coupling and phase coupling more than 99%, 95% and 95% of connections, respectively, were reliable for all frequencies.

### 2.9 Pattern similarity, inter-measure correlation and attenuation correction

We correlated the correlation patterns between different metrics, i.e. *AC*_*spur*_ vs. *AC*_*meas*_, *AC*_*spur*_ vs. *PC*, *AC*_*meas*_ vs. *PC*:

$$ic_{ACspur,ACmeas,i,s1,s2,f}=corr\left(P_{ACspur,i,s1,f},P_{ACmeas,i,s2,f}\right)$$

$$ic_{ACspur,PC,i,s1,s2,f}=corr\left(P_{ACspur,i,s1,f},P_{PC,i,s2,f}\right)$$

$$ic_{PC,ACmeas,i,s1,s2,f}=corr\left(P_{PC,i,s1,f},P_{ACmeas,i,s2,f}\right)$$

The inter-measure correlation *ic* between two metrics is defined as the Pearson correlation (*corr*) of the seed connectivity-patterns *P* at seed *i* and frequency *f* computed between different subjects *s1 ≠ s2*. The seed pattern *P* is defined as the connectivity of a given seed with the remaining 456 source points, i.e. one column in the full correlation matrix. We computed the inter-measure correlations *ic* for all 8930 unique subject pairings (95^2^-95), 457 connectivity patterns, 3 metric combinations, and 29 frequencies.

The measured inter-measure correlations do not only reflect the true underlying similarity of patterns but also the reliability with which these patterns are estimated. Measured correlation decreases with decreasing pattern reliability even if the true underlying pattern correlation remains identical (Fig. S2, dashed lines). This effect of reliability is known as attenuated correlations (Spearman, 1904). Following Spearman (1904), we corrected for this attenuation and normalized the mean inter-measure correlation *ic*_*M1,M2*_ by the pooled reliabilities within the measures *rel*_*M1*_ and *rel*_*M2*_.

$${\bar{ic}}_{M1,M2,i,f}=Z^{-1}\left(mean_{s}\left(Z\left(ic_{M1,M2,i,s1,s2,f}\right)\right)\right)$$

$${\bar{rel}}_{M1,i,f}=Z^{-1}\left(mean_{s}\left(Z\left(rel_{bs,M1,s1,s2,f}\right)\right)\right)$$

$${\bar{rel}}_{M2,i,f}=Z^{-1}\left(mean_{s}\left(Z\left(rel_{bs,M2,s1,s2,f}\right)\right)\right)$$

where the mean inter-measure correlation *ic* for different metric combinations *M1/M2* is defined as the mean over all possible subject combinations *s* with *s1 ≠ s2.* The same averaging is done for the reliabilities within the measures *M1* and *M2*. The function *Z* denotes the Fisher Z-transformation and *Z*^*−1*^ the inverse transformation:

$$Fisher^{’}s\;Z=\frac{1}{2}\ln\left(\frac{1+r}{1-r}\right)=\text{artanh}\left(r\right)$$

$$Fisher^{’}s\;Z^{-1}=r=\frac{e^{2Z}-1}{e^{2Z}+1}=\text{tanh}\left(Z\right)$$

Here, *ln* describes the natural logarithm, *artanh* the inverse hyperbolic tangent-, *tanh* the tangent function, *e* Euler’s number and *r* is the correlation coefficient. Finally, the attenuation corrected inter-measure correlation *icc* is defined as:

$$icc_{M1,M2,i,f}=\frac{{\bar{ic}}_{M1,M2,i,f}}{\sqrt{{\bar{rel}}_{M1,i,f}}\sqrt{{\bar{rel}}_{M2,i,f}}}$$

### 2.10 Simulation of attenuation corrected correlations

The attenuation corrected inter-measure correlation is unbiased. Accordingly, independent of the reliability, with which two patterns are measured, their expected attenuation corrected correlation is the true underlying correlation of these patterns. This is well illustrated by simulations (Fig. S2). We simulated two connectivity patterns of size n (n ​= ​45, approximating the effective degrees of freedom of the source-level MEG data; Hipp and Siegel, 2015) by drawing two times n data points from a normal distribution and applying the inverse Fisher’s Z transformation. The resulting nx2 matrix with both patterns is defined as P. From P we then computed P_r_ with a predefined correlation r between the two patterns. To this end, we defined the desired full correlation matrix *R* between the two patters as:

$$R=\begin{array}{cc} 1 & r\\ r & 1 \end{array}$$

Then, we applied the Cholesky factorization *chol* of *R* and multiplied P by the resulting matrix:

$$P_{r}=P*chol\left(R_{r}\right)$$

We simulated the two cases r ​= ​1 and r ​= ​0.3. Due to finite sampling the resulting correlation of the two vectors in P_r_ only approximates r. We therefore only used patterns for which the measured correlation coefficient differed from r by maximally 0.05. This threshold is necessary to not conflate the spread of the attenuation corrected correlation distributions with the variance of the simulated vector correlation, which otherwise grows towards r ​= ​0. We replicated the patterns for n ​= ​95 subjects and added inverse Fisher’s Z-transformed normally distributed noise independently to every subjects P_r_ with varying signal-to noise ratios: 0.5, 1, 2. Then, we computed the inter-measure correlation between patterns and the attenuation corrected correlation patterns and repeat the simulation 10,000 times for every SNR. The results are shown in Fig. S2.

### 2.11 Statistical testing of attenuation corrected correlations

A perfect attenuation corrected correlation (*icc = 1*) indicates that two cortical patterns are identical if there was perfect reliability. A value smaller than 1 indicates that there is a difference between the two patterns that cannot be explained by reduced reliability. Similarly a value different from 0 indicates pattern similarity. For statistical testing of *icc*, we applied leave-one-out Jackknifing and computed *icc* pseudo-values for each subject, source and frequency. We tested the generated pseudo-value distributions for normality using the Kolmogorov-Smirnov test. We then performed one-sided t-tests against 1 when appropriate. We corrected the resulting p-values with false-discovery rate correction across frequencies (Benjamini and Hochberg, 1995).

Notably, statistical testing re-introduces the reliability confound discussed above. While the mean pseudo-values of attenuation corrected correlations are independent of reliability, their variability across subjects increases with decreasing reliability (see also Fig. S2). This confound needs to be taken into account when interpreting the statistical significance.

## 3 Results

**Fig. 1:**
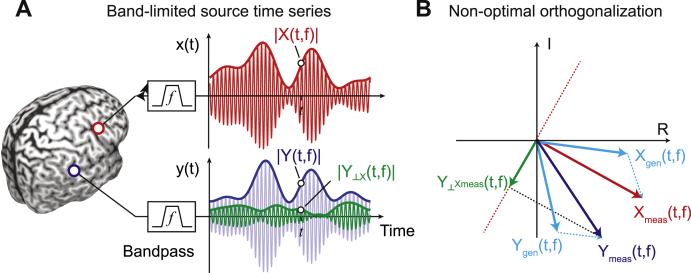
Principle of signal leakage reduction for amplitude relations (A) Illustration of band-limited time series from two sources X (red, upper panel) and Y (blue, middle panel) with their envelopes (thick lines). The green thick line resembles the envelope of signal Y orthogonalized on signal X. (B) illustrates how the orthogonalization can induce specious amplitude coupling in the presence of phase coupling and signal leakage. We orthogonalize the measures signal Y_meas_ onto the measured signal X_meas_. In the presence of signals leakage, both measured signals reflect a mix of the genuine signals X_gen_ and Y_gen_. For non-zero phase coupling between X_gen_ and Y_gen_, X_meas_ is rotated away from X_gen_. This causes sub-optimal signal orthogonalization and spurious amplitude coupling.

We quantified brain-wide neuronal phase- and amplitude-coupling from resting-state MEG measurements in 95 healthy participants. We applied source-reconstruction (Van Veen et al., 1997) to systematically characterize neuronal coupling at the cortical source level. Field spread (or signal leakage) can induce spurious coupling of sensor- and source-level MEG/EEG signals. Thus, we employed two coupling measures discounting signal leakage. We quantified phase-coupling using the weighted phase lag index (wPLI; Nolte et al., 2004; Vinck et al., 2011), which shows the best reliability of volume-conduction free phase-coupling measures (Colclough et al., 2016) and which, for the present results, showed the same coupling patterns as the imaginary coherence or the phase lag index (see Fig. S3 for a comparison of the wPLI with these other phase-coupling measures). For amplitude coupling, we employed pair-wise signal orthogonalization before estimating amplitude envelope-correlations (Fig. 1A) (Brookes et al., 2012; Hipp et al., 2012).

It has recently been shown that signal orthogonalization does not perfectly discount volume conduction in the presence of genuine phase coupling with non-zero phase delays (Palva et al., 2018). Intuitively, this is because, in the presence of signal leakage, such phase coupling systematically rotates the estimate of the signal to which one aims to orthogonalize, which results in sub-optimal orthogonalization and spurious amplitude-correlations (Fig. 1B). Thus, our first question was if the empirically measured amplitude-coupling patterns reflect this spurious amplitude coupling due to phase coupling. To test this, we directly estimated the spurious amplitude coupling with numerical simulations based on empirical parameters (see 2.6). In brief, for each subject, connection, and frequency, we simulated pairs of cortical signals with their signal leakage (resolution matrix), measured phase coupling (wPLI) and measured mean phase shift, but with no amplitude coupling. We then estimated the spuriously measured amplitude coupling for such signals. With this approach we computed the to be expected cortex-wide patterns of spurious amplitude coupling under the assumption of no genuine amplitude coupling.

### 3.1 Seed-based connectivity analysis

**Fig. 2:**
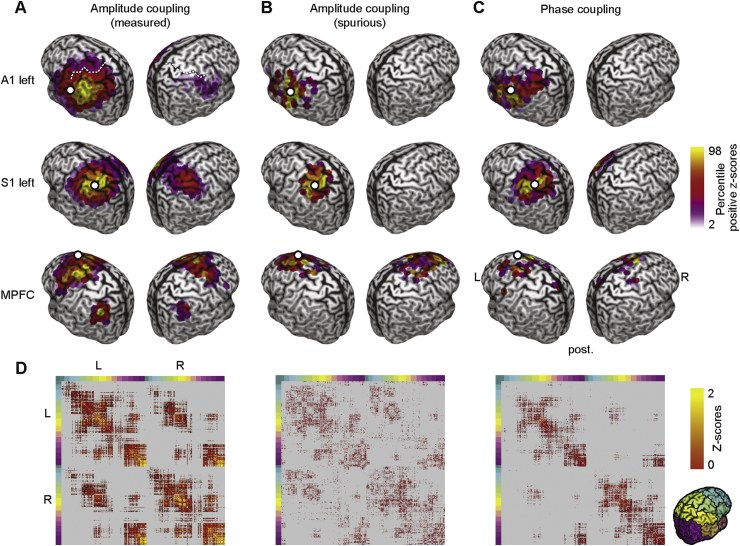
Seed based analysis for early sensory and higher order cortices at 16Hz Seed-based correlation structure (z-scores) of the left auditory (left A1, top row), left somatosensory (left S1, middle row), and the medial prefrontal cortex (MPFC, bottom row) for measured amplitude-coupling (A), spurious amplitude-coupling due to phase-coupling (B) and measured phase coupling (C). Coupling z-scores are tested against zero and statistically masked (p ​< ​0.05, FDR corrected). Color scale ranges from the 2nd to the 98th percentile of significant values, scaled within each panel. White dots indicate seed regions. The white dashed line in the top left panel highlights the central sulcus (see 4.3 for exact seed coordinates). (D) Full cortico-cortical connectivity at 16Hz. Seed-wise coupling z-scores are tested against zero and statistically masked (p ​< ​0.05, FDR corrected). Gray areas indicate non-significant connections. Colored marginals and the inset on the bottom right indicate the ordering of cortical seeds.

We started with a seed-based analysis (Fig. 2). We computed cortex-wide patterns of measured (Fig. 2A) and spurious (Fig. 2B) amplitude-coupling as well as phase-coupling (Fig. 2C) at 16 ​Hz for several early sensory and higher order cortical regions. As early sensory regions we chose the primary auditory (A1) and the somatosensory cortex (S1), which show strong inter-hemispheric connectivity and robust amplitude-coupling patterns at 16 ​Hz (Hipp et al., 2012; Mehrkanoon et al., 2014; Siems et al., 2016). For each seed, subject and both coupling modes, we z-scored the raw coupling measures and tested for z-scores larger than zero across subjects (one-sided *t*-test, FDR-corrected). This revealed which connections showed significant above-average coupling, discounting global offsets of coupling measures (Hipp et al., 2012).

For both sensory seeds (A1 and S1), amplitude coupling was strongest to regions surrounding the seed region and to the homologous area in the other hemisphere. Phase coupling did not show this pattern, but only above-average connectivity surrounding the seed. Similarly, spurious amplitude coupling was restricted to regions surrounding the seed.

Our findings for a higher order seed region confirmed these results. We investigated phase and amplitude coupling for the medial prefrontal cortex (MPFC, Fig. 2A–C bottom row), which shows a complex connectivity structure for amplitude coupling at 16 ​Hz (Hipp et al., 2012; Siems et al., 2016). We found that amplitude coupling of MPFC peaked bilaterally in the dorsal prefrontal and lateral parietal cortices. In contrast, phase coupling and spurious amplitude coupling only peaked surrounding the seed region.

We extended our analysis to the entire correlation matrix at 16 ​Hz (Fig. 2D). The results confirm the observations from the seed-based analyses. In comparison to phase coupling and spurious amplitude coupling, measured amplitude coupling displayed the most pronounced interhemispheric connectivity.

### 3.2 Genuine amplitude coupling

**Fig. 3:**
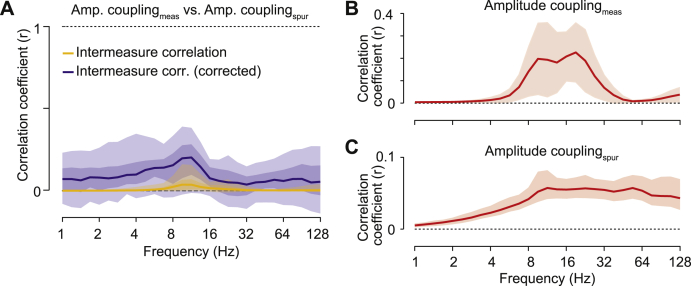
Correlation between measured and spurious amplitude-coupling patterns (A) Frequency resolved correlation between measured and spurious amplitude-coupling patterns. Lines indicate median attenuation corrected (blue) and uncorrected (yellow) correlation. Shaded areas indicate the 5–95% and 25–75% inter-percentile range across cortical space. (B) Reliability, i.e. correlation, of measured amplitude-coupling patterns between subjects. (C) Reliability of spurious amplitude-coupling patterns between subjects. Shaded areas indicate the 5– 95% interquantile range.

To quantitatively address our first main question, i.e. if the measured amplitude coupling reflects genuine amplitude coupling, we systematically assessed the similarity of the cortical patterns of spurious and measured amplitude coupling across frequencies (Fig. 3).

For each frequency and both measures, we computed the coupling between all cortical regions, i.e. we computed the full connectivity matrices of the cortex-wide measured and spurious amplitude coupling (as shown in Fig. 2D for 16 ​Hz). We then correlated the patterns of spurious and measured amplitude coupling for each cortical seed region (3 examples from the 457 sources in one frequency are shown in Fig. 2). In other words, we correlated each column of the connectivity matrices between measures. Averaged across all seed regions, for all frequencies, this revealed a very low correlation between spurious and measured amplitude coupling patterns with median correlation coefficients below 0.05 (Fig. 3A, yellow line).

At first sight, the low correlation between measured and spurious amplitude-coupling patterns suggests that there is indeed genuine amplitude coupling. However, it is important to realize that the correlation between two metrics does not only reflect their true underlying correlation, but also the metrics’ reliability (Bergholm et al., 2010; Hipp et al., 2012; Siems et al., 2016; Spearman, 1904). A reduced reliability of two measures, e.g. due to noise, leads to a lowered measured correlation even if the true underlying correlation between the two measures is higher (Fig. S2, dashed lines). Thus, the observed low and frequency specific correlation between spurious and measured amplitude-coupling patterns may merely reflect the low reliability of either measure, and thus, does not allow for directly inferring genuine amplitude coupling.

We applied attenuation correction of correlations (Hipp and Siegel, 2015; Siems et al., 2016; Spearman, 1904) to account for the effect of signal reliability. Attenuation corrected correlations quantify how strong a correlation would be for perfectly reliable signals (Fig. S2). We employed the between-subject correlation of the measured and spurious amplitude coupling-patterns as a proxy for each measure’s reliability. For the measured amplitude coupling, between-subject reliability peaked around 16 ​Hz (Fig. 3B) compatible with previous findings (Hipp and Siegel, 2015; Siems et al., 2016). For the spurious amplitude-coupling, reliability was overall lower and decreased for frequencies below 16 ​Hz (Fig. 3C).

We corrected the correlation between measured and spurious amplitude-coupling patterns for these reliabilities by pooled division (see 2.8 and 2.9) (Fig. 3A, blue line). As predicted, the overall correlation between measured and spurious amplitude-coupling patterns increased. However, the median attenuation corrected correlation remained low between 0.05 and 0.19 for all frequencies. We statistically assessed if there was indeed spurious amplitude coupling contributing to the measured amplitude coupling, i.e. if the attenuation corrected correlations between measured and spurious amplitude-coupling patterns were significantly different from 0. Attenuation corrected correlation is an unbiased estimate (Fig. S2). Thus, we applied a leave-one-out jackknifing procedure and false-discovery rate correction (Benjamini and Hochberg, 1995). Across the entire spectrum, we found that less than 1% of the seed patterns showed significant (p ​< ​0.05, corrected) correlations with spurious amplitude coupling patterns. On average across all frequencies and cortical seeds, less than 2% of the variance in the measured amplitude-coupling patterns could be explained by spurious amplitude coupling.

The above simulations may not capture all non-linearities in the relationship of phase coupling, amplitude coupling and the spurious amplitude coupling. Thus, we repeated our analysis using a non-parametric rank correlation that is insensitive to monotonous non-linearities (Fig. S4). The results of this control analysis were nearly identical to the results based on Pearson’s correlation. Taken together, we concluded that, for the data at hand, the effect of spurious amplitude coupling on measured amplitude coupling patterns was small.

**Fig. 4:**
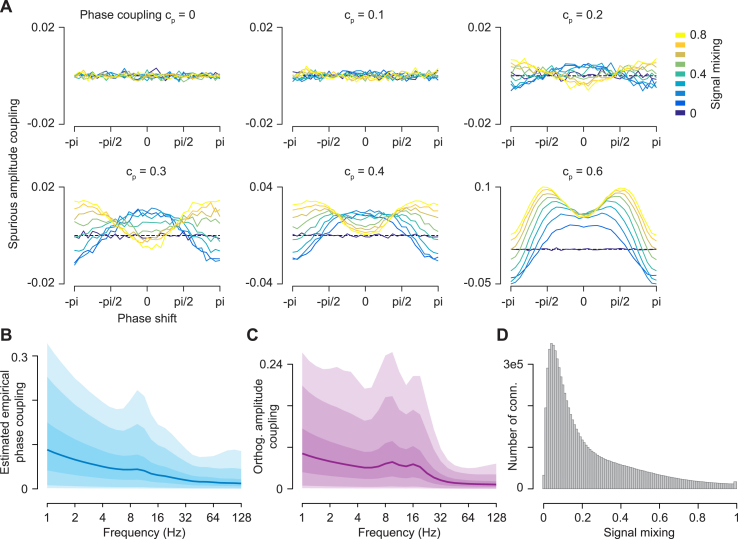
Systematic simulation of spurious amplitude coupling and empirical parameters (A) Simulation of spurious amplitude coupling as function of phase coupling (panels), phase shift between signals (-pi to pi) and signal mixing (colored lines). (B) Distribution of the estimated empirical phase coupling (C) Distribution of the measured amplitude coupling (D) Distribution of empirical signal mixing. All shaded areas indicate the 1–99%, 5–95% and 25–75% interquantile ranges.

Why does the spurious amplitude show little effect on the measured amplitude coupling? We speculated that this might be due to the dynamic range of empirical phase-coupling. To investigate this, we performed systematic simulations across a broad set of coupling parameters and mixing (compare Palva et al., 2018) (Fig. 4A). These simulations showed that only for substantial phase coupling (*c*_*p*_ >= 0.2) the relation between phase-shift, phase coupling, mixing and spurious amplitude coupling appears stable (Fig. 4A). However, the estimated empirical phase coupling was mostly below this range (Fig. 4B). The median over all frequencies was between 0.01 and 0.09. For all frequencies, but the lowest two, phase coupling was below 0.2 and 0.3 for more than 95% and 99% of the connections, respectively (Fig. 4B). Complementary, spurious amplitude coupling values approached the levels of measured amplitude coupling (Fig. 4C) only at higher phase coupling (*c*_*p*_ >= 0.4) at a phase shift around 90° (Fig. 4A). Thus, for the present data, the observed small effect of spurious amplitude coupling is likely due to the small empirical phase coupling.

### 3.3 Comparing amplitude-coupling and phase-coupling networks

The above results suggest that the measured amplitude coupling patterns are dominated by genuine amplitude coupling. This allowed us to address our second main question: Are amplitude- and phase coupling-patterns of cortical connectivity different?

**Fig. 5:**
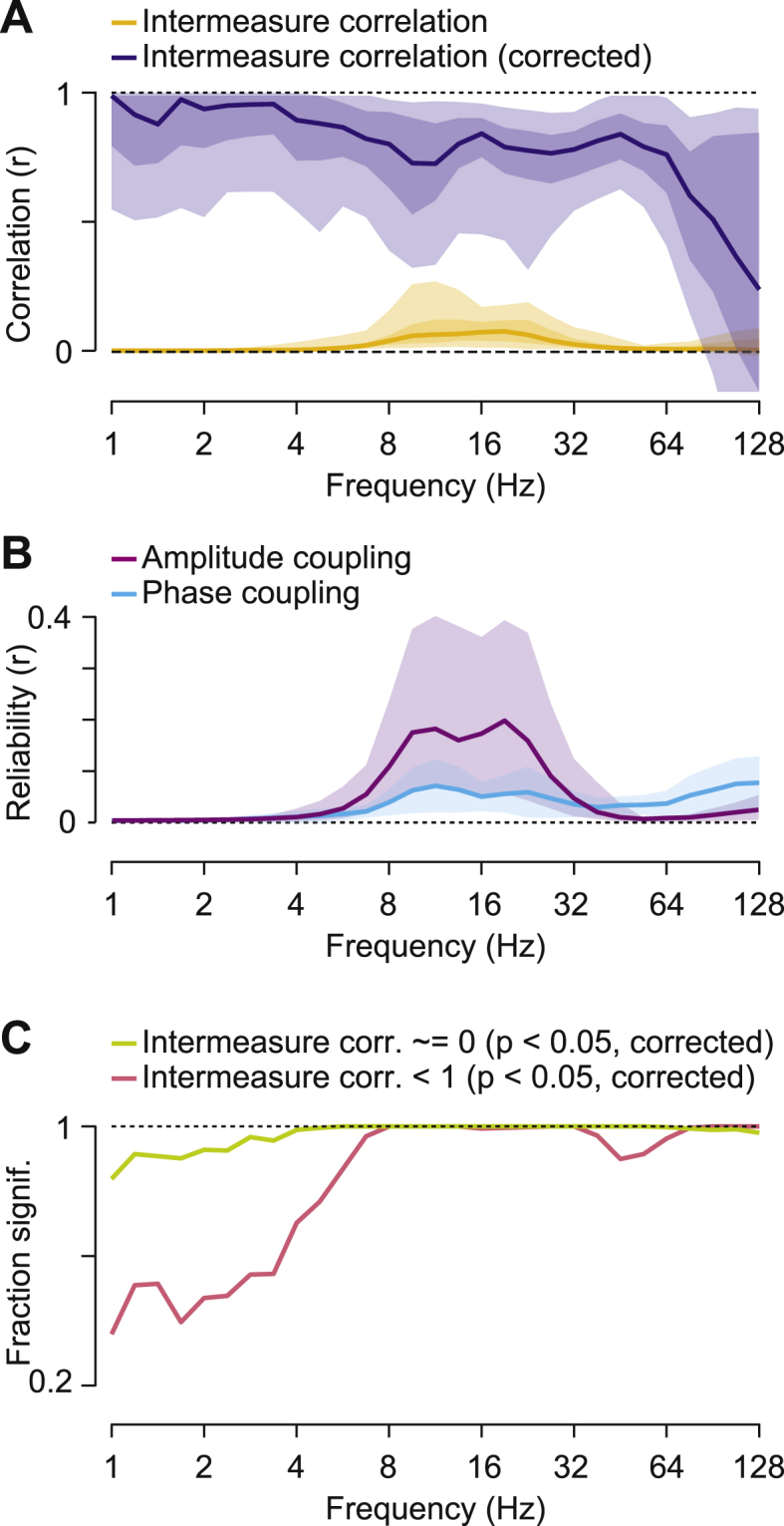
Correlation between amplitude- and phase coupling patterns (A) Spectrally resolved distribution of the correlation between corrected amplitude- and phase coupling patterns. The lines show the median of the uncorrected (yellow) and attenuation corrected (blue) correlations. The shaded areas indicate the 5–95% and 25–75% interquantile range across cortical space. (B) Between-subject reliability of corrected amplitude coupling (purple) and phase coupling (cyan) as a function of frequency. Shaded areas indicate the 5–95% interquantile range across cortical space. (C) Spectrally resolved fraction of patterns that show an attenuation corrected correlation significantly different from 0 (green line) or smaller than 1 (red line) (p ​< ​0.05, FDR-corrected).

To address this question, we partializing out (linear regression) the spurious amplitude coupling patterns from the measured amplitude coupling patterns (see Fig. S5 for example seed patterns). We then correlated the resulting corrected amplitude coupling patterns with the measured phase coupling patterns for every seed pattern and frequency. We found highest correlations from 8 to 26 ​Hz (Fig. 5A yellow line). As for the correlation between spurious and measured amplitude coupling patterns above, these correlations are attenuated by measurement reliability (Fig. 5B and S2). We therefore computed the attenuation corrected correlation between the corrected amplitude coupling and phase coupling patterns (Spearman, 1904).

The attenuation correction substantially increased the correlation, i.e. similarity between the two coupling modes (Fig. 5A blue line). The median corrected correlation was around 0.8. The spectral distribution indicated three regimes: Very high similarity for frequencies below 4 ​Hz, a lower similarity from 5 to 64 ​Hz and very high variability of similarity above 64 ​Hz. We statistically tested if the two coupling modes shared pattern similarities, i.e. if the correlation was significantly different from 0. Indeed, for the entire spectrum and almost all seed patterns there was significant similarity between amplitude- and phase coupling patterns (Fig. 5C green line; p ​< ​0.05 corrected). However, even though the similarity was high, we found that the patterns were not identical. For almost all seed patterns at frequencies above 4Hz pattern similarity was significantly smaller than 1 (Fig. 5C red line; p ​< ​0.05 corrected). Thus, amplitude and phase coupling patterns were similar but not identical. Again, we repeated the correlation analysis based on rank-correlation (Fig. S6). This yielded almost identical results suggesting that the dissimilarities were not driven by monotonous non-linear interactions between amplitude- and phase-coupling.

**Fig. 6:**
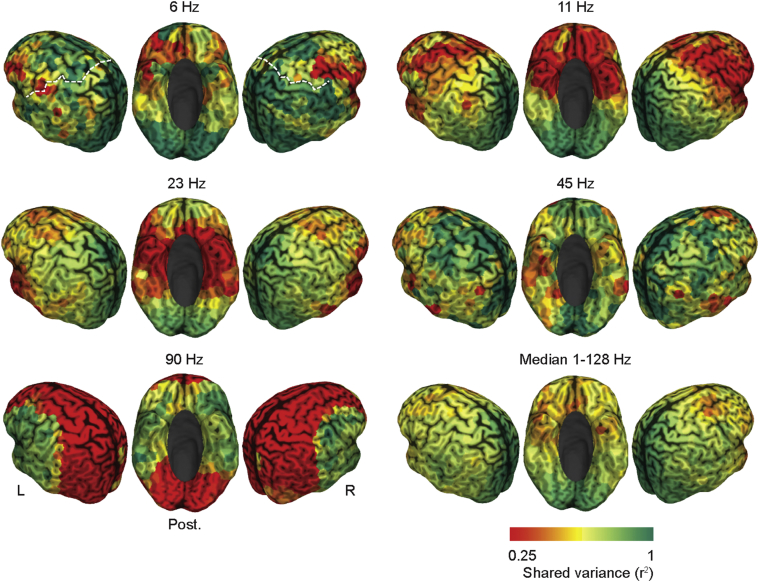
Cortical distribution of the correlation between amplitude- and phase coupling patternsCortical distribution of the shared variance (r^2^) between corrected amplitude- and phase coupling patterns for 6 ​Hz, 11 ​Hz, 23 ​Hz, 45 ​Hz and 90 ​Hz. The bottom right panel shows the median across all assessed frequencies (1–128Hz). Red areas indicate differences whereas green areas indicate similarity between coupling modes. The white dashed line (top left panel) indicates the central sulcus.

The average attenuation corrected correlations did not show strong spectral differences (Fig. 5A). However, the cortical distribution of connectivity patterns that are most dissimilar between coupling modes may be frequency specific. Indeed, for all investigated frequencies, we found cortical regions with significantly dissimilar amplitude- and phase-coupling patterns (Fig. 5C) and less than 25% shared variance between coupling patterns (Fig. 6). The median pattern across all frequencies (Fig. 6 bottom right) displayed the strongest differences bilaterally in lateral prefrontal, orbitofrontal, anterior temporal and temporo-parietal areas. The lateral prefrontal differences appear to be strongest in the theta to alpha frequency range (6–11 ​Hz) and the temporal differences in the beta (23 ​Hz) and low gamma frequencies (45 ​Hz; Fig. 6).

**Fig. 7:**
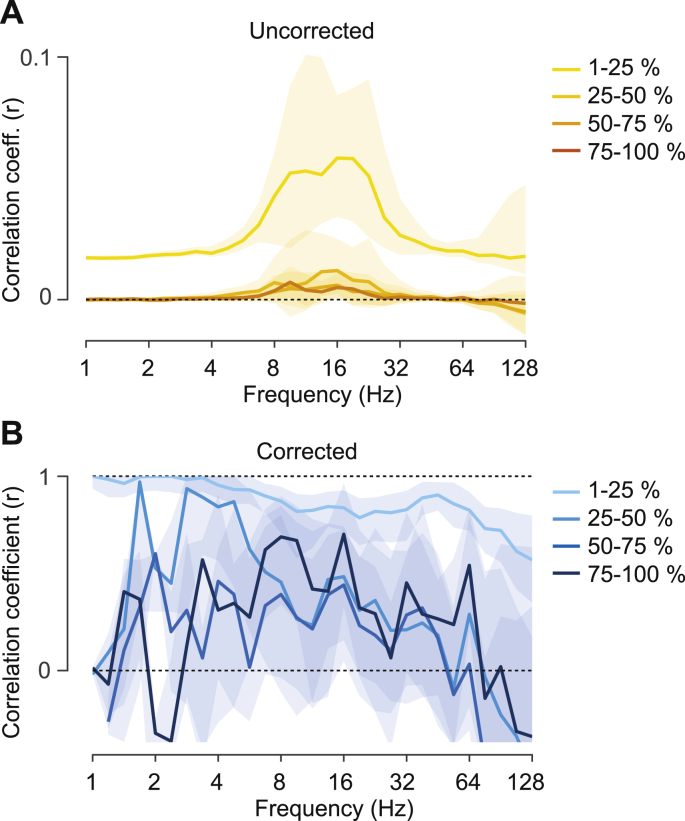
Correlation between amplitude- and phase coupling patterns as a function of connection distanceSpectrally resolved correlation between corrected amplitude- and phase coupling patterns for different regimes (quartiles) of Euclidean distance between the cortical seeds of each connection. (A) Shows the uncorrected and (B) the attenuation corrected correlation between corrected amplitude coupling and phase coupling patterns. Shaded areas indicate the 25–75% interquartile range over space.

Which features of connectivity drive these differences between coupling modes? Given the higher complexity of amplitude coupling patterns as compared to phase-coupling patterns (Fig. 2A & Fig. S5A), we investigated the relation between amplitude and phase coupling patterns as a function of spatial distance. We split all cortico-cortical connections into 4 quartiles and, for each quartile, repeated the correlation of connectivity patterns (Fig. 7). We found that patterns were most similar for short distance connections and were more dissociated for longer connections (Fig. 7B). Thus, the differences between amplitude- and phase-coupling patterns were mostly driven by differences of long-distance connectivity.

## 4 Discussion

Our results provide, to our knowledge, the first systematic comparison of cortical phase- and amplitude-coupling patterns in the human brain. We found similarities and differences between both coupling modes that were widely distributed across frequencies and the entire cortex. By combining empirical measurements and simulations we showed that the observed differences were not caused by known methodological biases, but instead reflect a genuine dissociation between coupling modes. The observed differences suggest that the two coupling modes may at least partly reflect distinct neural mechanisms. Furthermore, our results highlight and clarify the compound nature of amplitude coupling measures applied to orthogonalized signals.

### 4.1 Discounting confounding factors

Our analyses discount three critical factors that confound the estimation of neuronal coupling patterns and their comparison. First, we employed amplitude correlations of orthogonalized signals (Brookes et al., 2012; Hipp et al., 2012) and the weighted phase-lag index (Vinck et al., 2011). Using these coupling measures ensured that the measured coupling did not reflect spurious coupling due to field-spread.

Second, recent studies suggest that amplitude correlation between orthogonalized signals is a compound measure, which is affected by the phase-coupling between those signals (Palva et al., 2018). We estimated the spurious amplitude coupling due to phase-coupling using simulations based on the empirically measured phase-coupling. We then partialized the resulting spurious amplitude-coupling patterns from the measured amplitude-coupling patterns.

Third, for the comparison between coupling modes, we employed attenuation correction of correlations (Spearman, 1904). This approach allows correcting for the attenuation of measured correlation caused by sub-optimal measurement reliability. Attenuation correction of correlations is a powerful analytical approach that has been successfully employed before to compare MEG with fMRI (Hipp and Siegel, 2015) and MEG with EEG (Siems et al., 2016). Importantly, reliability in the present study refers to the stability of coupling patterns across subjects, which effectively takes into account all sources of variance across subjects, including measurement and finite-sampling noise, noise caused by neural activity not of interest, and inter-subject variability. The employed approach corrects for all these sources of variance, which attenuate measured correlations and may thus induce spurious spectral and spatial specificity.

Our results indicate that the raw correlation between amplitude- and phase coupling patterns is strongly affected by measurement reliability. Attenuation correction suggests that the peaked raw correlation around 16 ​Hz reflects the strength of intrinsic cortical rhythms around this frequency, rather than a frequency specific relation of the two coupling modes (compare also Zhigalov et al., 2017).

### 4.2 Phase-coupling sensitivity of orthogonalized amplitude correlation

Our results provide a critical reassessment of well-established amplitude-coupling measures of orthogonalized signals (Brookes et al., 2012; Hipp et al., 2012). It has recently been pointed out that, in the presence of field-spread, these measures are sensitive to phase coupling with non-zero phase lag (Palva et al., 2018). Here, we combined the simulation approach put forward by Palva et al. (2018) with empirical measurements to systematically evaluate the sensitivity of these measures to phase coupling across the human cortex.

The bias of orthogonalized amplitude coupling measures left open the possibility that the described amplitude coupling patterns (Brookes et al., 2012; Hipp et al., 2012) merely reflect phase coupling in combination with field spread. Our results provide several lines of evidence against this hypothesis.

First, the spurious amplitude-correlation showed little consistent connectivity, whereas the measured amplitude-coupling patterns showed complex and multimodal distributions (Fig. 2). Second, the between subject reliability of coupling patterns clearly dissociated measured and spurious amplitude coupling (Fig. 3B and C). Third, for all frequencies and cortical regions spurious amplitude coupling patterns could not explain more than 10% and on average less than 4% of the variance in measured amplitude-coupling patterns (Fig. 3A). In sum, our findings suggest that for the present data the magnitude of spurious amplitude coupling and its effects on measured amplitude coupling is small.

Nevertheless, it is important to highlight the compound nature of amplitude correlations of orthogonalized signals. Our simulations confirm that phase coupling, phase shift and mixing have a marked effect on the amount of spurious amplitude coupling (Fig. 4A). In accordance with Palva et al. (2018), our results show that this compound nature needs to be taken into account in particular for cases with high or variable phase-coupling.

### 4.3 Relation between phase- and amplitude coupling

Our results show that amplitude- and phase coupling patterns bear substantial similarities (Fig. 5). These similarities may result from one or more common underlying neural mechanism. Synaptic interactions between neuronal populations may induce both, coupling of phases and amplitudes of these neuronal populations. Similarly, common input to neuronal populations will co-modulate and thus couple both, phases and amplitudes (Tewarie et al., 2018).

Alternatively, also causal relations between both coupling modes may result in correlations. For example, phase-locking may enhance neuronal interactions, and thereby, enhance amplitude coupling (Fries, 2015; Womelsdorf et al., 2007).

Despite the high attenuation corrected correlation between amplitude- and phase coupling patterns we found that amplitude and phase-coupling patterns are not identical. Which factors may cause the observed differences between phase- and amplitude coupling patterns (Fig. 5)?

First, different non-linearities between coupling modes may induce differences. The same underlying neuronal interaction or common input may have different effects on both coupling modes. However, in contrast to our present results, this effect should be spectrally and spatially unspecific. Furthermore, our present results were robust to using a non-parametric correlation insensitive to monotonous non-linearities (Fig. S5). These results suggest that such non-linearities cannot explain the observed differences between coupling modes.

Second, distinct neuronal mechanisms may underlie the two coupling modes. On the one hand, for example, neuromodulation may co-modulate the amplitude of rhythms in different brain regions (van den Brink et al., 2019). Or, as recently proposed, slow fluctuations of extracellular potassium concentrations and structural connectivity may drive long-range power co-fluctuations (Krishnan et al., 2018). These mechanisms may induce amplitude coupling on a slow temporal scale without necessarily causing phase coupling. On the other hand, synaptic interactions triggered by intrinsic activity or sensory inputs may induce phase-coupling between areas without driving identical amplitude co-modulations (Landau et al., 2015). The notion of different mechanisms underlying both coupling modes is also supported by their distinct temporal dynamics (Daffertshofer et al., 2018).

Notably, also residual non-neuronal signals, such as e.g. muscle activity, eye-movements or cardiac activity, might systematically affect both coupling modes. Such sources may induce or hamper reliable coupling patterns across subjects and coupling modes, and thereby, affect the correlation of coupling patterns between coupling modes.

### 4.4 Functional role of coupling modes

Phase-coupling of neuronal population may regulate their interactions by aligning rhythmic excitability fluctuations and rhythmic inputs (Fries, 2015). Similarly, amplitude-coupling may modulate interactions by temporally aligning processing associated with low or high oscillatory amplitudes across brain regions (Siegel et al., 2012; von Nicolai et al., 2014). While the observed differences between coupling modes may reflect such functional roles, the present results hold independent from such potential functions. In fact, even if phase- or amplitude coupling merely reflect neural interactions without a causal mechanistic role, our results show that these coupling modes provide partially dissociated and thus non-redundant information about neuronal interactions. This suggests that both coupling modes provide complimentary information on large-scale neuronal interactions during cognitive processes and on their alteration in neuropsychiatric diseases.

### 4.5 Limitations

Our results are based on the assumption of Gaussian signals. Deviations from this assumption may have an effect on two levels. First, non-Gaussian signals will lead to sub-optimal orthogonalization (Brookes et al., 2014, 2012; Hipp et al., 2012). Second, the spurious amplitude coupling of non-Gaussian signals will deviate from the simulated estimates based on Gaussian signals. Thus, optimal estimation of the spurious amplitude coupling requires a further systematic assessment of the effect of signal distributions.

### 4.6 Future directions

Our results provide a critical first step to unravel the relationship between neuronal phase- and amplitude-coupling. Further invasive studies are needed to investigate this relationship and the underlying mechanisms on the cellular and circuit level, as well as to link the present results to spiking activity of individual neurons. Additionally, the investigation of non-linear and cross-frequency relationships, i.e. phase- and amplitude-coupling across different frequencies (Brookes et al., 2016; Diekelmann and Born, 2010; Mandke et al., 2018; Schroeder and Lakatos, 2009; Tewarie et al., 2016; von Nicolai et al., 2014; Womelsdorf et al., 2007) as well as the application of directed interaction measures (Hillebrand et al., 2016; Lobier et al., 2014; Vinck et al., 2015) may allow identifying generic links between the coupling modes.

## Author contributions

Both authors conceived the study, wrote and revised the paper. M.S.1 performed the data analysis.

## CRediT authorship contribution statement

**Marcus Siems:** Writing - original draft, Formal analysis. **Markus Siegel:** Writing - original draft.
