# Supplementary material for: Dissociated neuronal phase- and amplitude-coupling patterns in the human brain
Source: Neuroimage. 2020 Apr 1;209:116538. doi: 10.1016/j.neuroimage.2020.116538 (PMC7068703; doi:10.1016/j.neuroimage.2020.116538)
Supplement: Multimedia component 1 [file mmc1.pdf]

Supplementary Figures

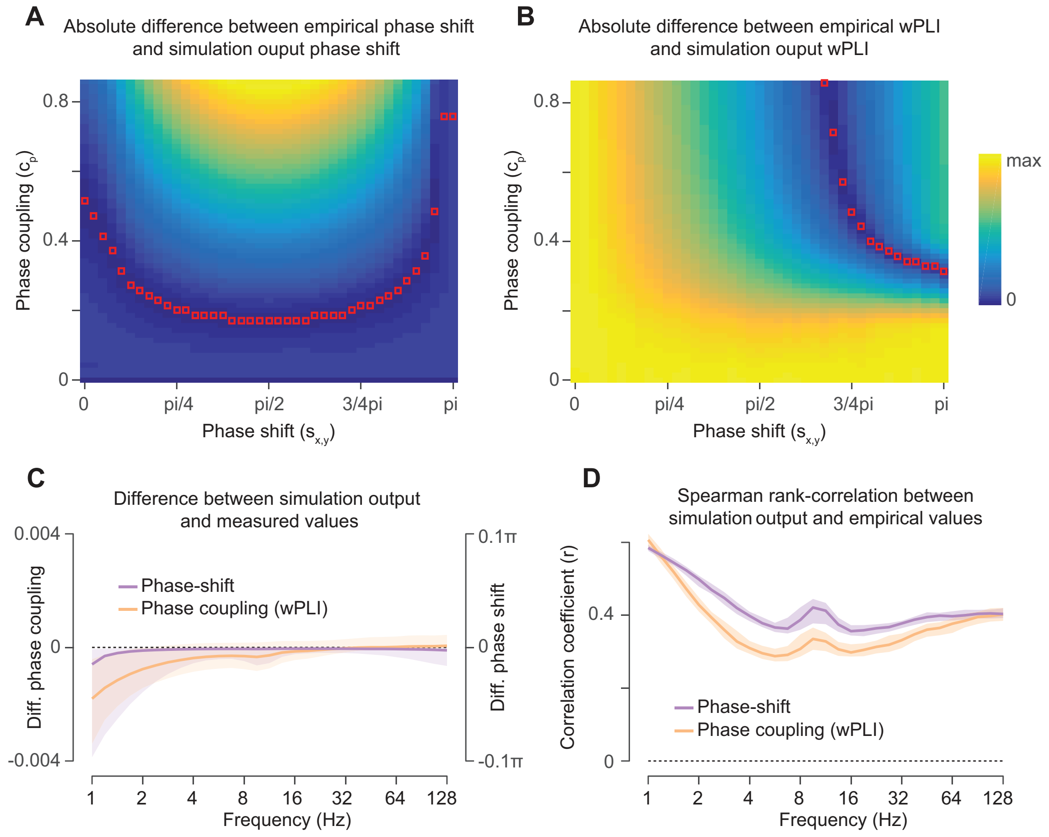

Figure S1. Assessment of parameter estimation

Example distribution (at measured signal mixing of 0.2) of the difference between simulation output and empirically measured (A) phase-shift and (B) phase coupling as a function of simulation input (x-axis phase-shift, y-axis phase coupling parameter). Red squares indicate the zero contour-line of the minimum difference between simulation output and empirically measured values as a function of input phase-shift and phase-coupling. We chose the intersection between these isolines as the estimated empirical phase shift and phase coupling for the signal construction to obtain simulation outputs with the measured wPLI and phase shift. (C) Difference between the optimal simulation output and the empirically measured values. Shaded areas indicate the 5-95% interquantile range across subjects and connection space. (D) Rank correlation between the optimal simulation output and the empirically measured values. Shaded areas indicate the 25-75% interquartile range.

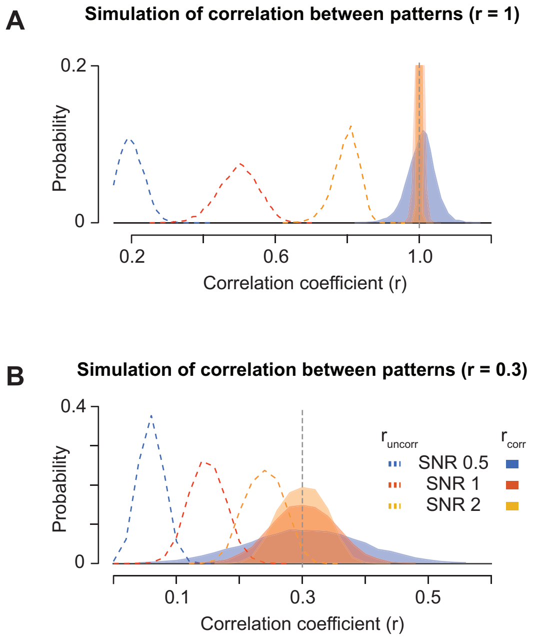

Figure S2. Simulation of attenuation corrected and uncorrected pattern correlation as a function of ground-truth pattern correlation and between-subject noise

Distribution of simulated attenuation corrected (solid lines) and uncorrected correlations (dashed lines) between perfectly correlated patterns (A) and patterns correlated at r=0.3 (B). We added uncorrelated noise to the patterns: The signal-to-noise ratio (SNR) levels are set to 0.5, 1 and 2 (for further details see 2.10). The y-axis in (A) is trimmed to 0.2 for visualization purposes: maximum values for SNR 1 = 0.4 and SNR 2 = 0.83.

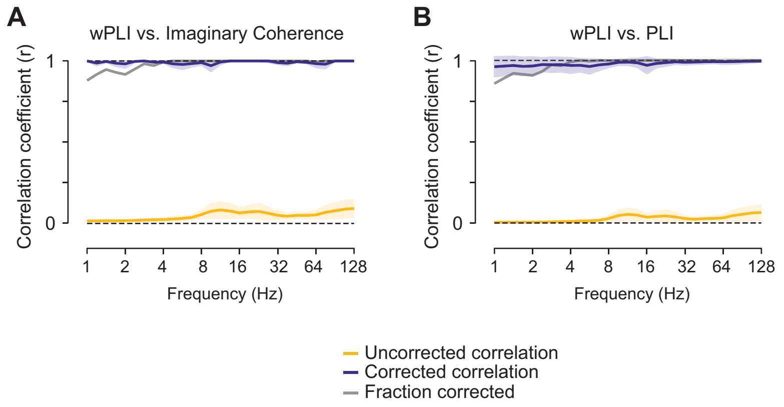

Figure S3. Comparison between phase-coupling measures

Distribution of attenuation corrected (blue) and uncorrected (yellow) correlation between seed-patterns between the weighted phase lag index (wPLI) and imaginary coherency (ImC) (A) and between the wPLI and phase lag index (PLI) (B). The gray line indicates the relative number of corrected seed patterns. Shaded areas indicate the standard deviation across cortical space.

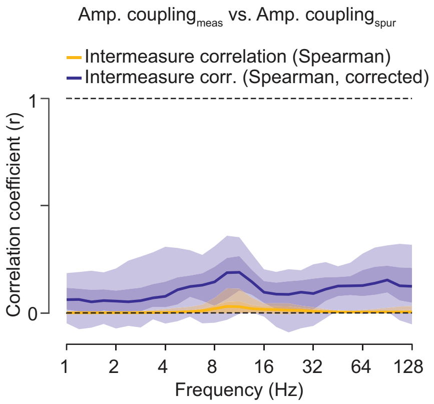

Figure S4. Non-parametric correlation between spurious and measured amplitude coupling patterns

Lines indicate median attenuation corrected (blue) and uncorrected (yellow) correlation. Shaded areas indicate the 5-95% and 25-75% inter-percentile range over space.

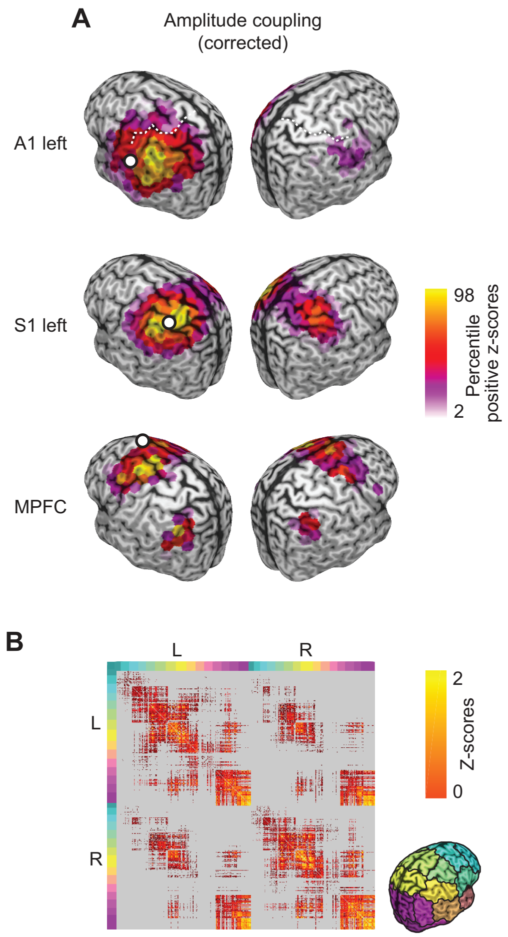

Figure S5. Corrected amplitude coupling patterns

(A) Seed-based correlation structure (z-scores) of the left auditory (left A1, top row), left somatosensory (left S1, middle row), and the medial prefrontal cortex (MPFC, bottom row) for the corrected amplitude-coupling at 16 Hz. Coupling z-scores are tested against zero and statistically masked ($p<0.05$, FDR corrected). Color scale ranges from the 2nd to the 98th percentile of significant values, scaled within each panel. White dots indicate seed regions. The white dashed line in the top left panel highlights the central sulcus (see 4.3 for exact seed coordinates). (B) Full cortico-cortical connectivity at 16Hz. Seed-wise coupling z-scores were tested against zero and statistically masked ($p<0.05$, FDR corrected). Gray areas indicate non-significant connections. Colored marginals and the inset on the bottom right indicate the ordering of cortical seeds.

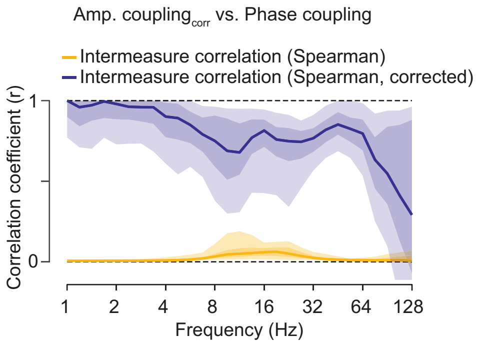

Figure S6. Non-parametric correlation between corrected amplitude coupling and phase coupling patterns

Lines indicate median attenuation corrected (blue) and uncorrected (yellow) correlation. Shaded areas indicate the 5-95% and 25-75% inter-percentile across cortical space.
